# Tirofiban preserves platelet loss during continuous renal replacement therapy in a randomised prospective open-blinded pilot study

**DOI:** 10.1186/cc6998

**Published:** 2008-08-29

**Authors:** Andreas Link, Matthias Girndt, Simina Selejan, Ranja Rbah, Michael Böhm

**Affiliations:** 1 Klinik für Innere Medizin III, Universitätsklinikum des Saarlandes, Kirrberger Strasse, 66421 Homburg/Saar, Germany; 2 Klinik für Innere Medizin IV, Universitätsklinikum des Saarlandes, Kirrberger Strasse, 66421 Homburg/Saar, Germany

## Abstract

**Introduction:**

Approximately one third of all patients with cardiogenic shock suffer from acute kidney injury. Percutaneous coronary intervention, intra-aortic balloon pump, and continuous renal replacement therapy (CRRT) require effective antiplatelet therapy and anticoagulation, resulting in a high risk for platelet loss and bleeding events. The reversible platelet glycoprotein IIb/IIIa receptor inhibitor tirofiban was investigated to preserve platelet number and activation in a prospective open-blinded endpoint evaluation study.

**Methods:**

Forty patients with cardiogenic shock and acute kidney injury requiring CRRT were randomly assigned to two groups receiving unfractioned heparin (UFH) (n = 20) or a combined anticoagulation with UFH and tirofiban (n = 20). The primary endpoint was platelet loss during CRRT. Secondary endpoints were urea reduction, haemofilter life span, bleeding events, and necessity for platelet transfusions.

**Results:**

In UFH-treated patients, the percentage of platelet-monocyte aggregates significantly increased (*P *< 0.001) and consecutively platelet cell count significantly decreased (*P *< 0.001). In contrast, combined treatment with UFH and tirofiban significantly decreased platelet-monocyte aggregates and platelet numbers (*P *< 0.001).

**Conclusions:**

This pilot study provides evidence that the use of tirofiban in addition to UFH prevents platelet loss and preserves platelet function in patients with cardiogenic shock and acute kidney injury requiring CRRT. The pathophysiological inhibition of platelet aggregation and platelet-monocyte interaction appears to be causally involved.

## Introduction

Approximately one third of all patients with cardiogenic shock suffer from acute kidney injury. This increases in-hospital mortality from 53% to 87% [1]. Early revascularisation, intra-aortic balloon pump (IABP), and antithrombotic therapy improve outcomes in cardiogenic shock [2]. In cases of acute kidney injury with necessity for continuous renal replacement therapy (CRRT), effective anticoagulation is required. However, excessive anticoagulation in critically ill patients receiving CRRT may cause changes in platelet function, platelet loss, and bleeding events [3,4].

The contact of blood with surfaces of the extracorporeal membrane circuits and different anticoagulants leads to platelet and leukocyte activation [5,6] and platelet-leukocyte coaggregation [7,8]. All of these interactions result in glycoprotein (GP) IIb/IIIa receptor activation that becomes capable of binding soluble fibrinogen [9]. GP IIb/IIIa receptor antagonists primarily act on the platelet surface by inhibition of fibrinogen binding that is essential for platelet bridging and aggregate formation [10].

Tirofiban is a reversible short-acting inhibitor of platelet GP IIb/IIIa receptors used in acute coronary syndromes and cardiac interventions [11]. The hypothesis that tirofiban preserves platelet number and function and shortens postoperative bleeding times was first described in baboons [12] and in patients with heparin-induced thrombocytopenia type II (HIT-II) during cardiopulmonary bypass surgery [13,14]. The aim of this study was to prove the efficacy of tirofiban on platelet protection and safety in critically ill patients with cardiogenic shock and necessity for CRRT receiving either conventional therapy with unfractionated heparin (UFH) or additional tirofiban.

## Materials and methods

**Figure 1:**
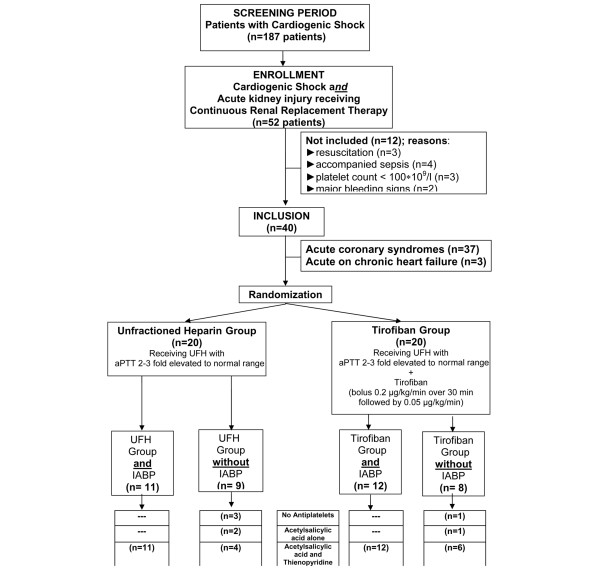
Study flowchart. Patients were randomly assigned in different anticoagulation regimens (unfractioned heparin [UFH] versus UFH + tirofiban), separated according to the concomitant therapy with or without intra-aortic balloon pump (IABP). Furthermore, the concomitant antiplatelet therapy and the number of patients included in each subgroup were added. Exclusion criteria include cardiopulmonary resuscitation, suspected concomitant sepsis defined by haemodynamic criteria (reduced systemic vascular resistance), a platelet count of less than 100 × 10^9^/L, or major bleeding signs (one patient retroperitoneal and one patient gastric haemorrhage). aPTT, activated partial thromboplastin time.

The PROBE (prospective randomised open-blinded endpoint) design study was approved by the ethics committee of the state medical board. Patients with cardiogenic shock (n = 187) and acute kidney injuries with necessity for CRRT (n = 52) were evaluated from January 2006 to December 2007. Cardiogenic shock was confirmed by both clinical and haemodynamic criteria. The clinical criteria were hypotension (systolic blood pressure of less than 90 mm Hg for at least 30 minutes or the need for supportive vasoactive medications to maintain a systolic blood pressure of greater than 90 mm Hg) and evidence of end-organ hypoperfusion (cool, diaphoretic extremities). Haemodynamic criteria were a reduced cardiac index (<2.2 L/minute per m^2^) and the presence of elevated pulmonary capillary occlusion pressure (>15 mm Hg) [15]. Acute kidney injury with necessity for CRRT was defined as a urine output of less than 0.5 mL/kg per hour for 6 hours and/or an increase in serum creatinine of greater than or equal to 1.5 mg/dL within 24 hours according to the RIFLE (Risk, Injury, Failure, Loss, and End-stage kidney disease) criteria grade risk of renal dysfunction [16]. After admission to the intensive care unit (ICU) and after informed consent was given, all study participants (n = 40) were randomly assigned using a computer algorithm: UFH (n = 20) versus UFH + tirofiban (n = 20). Figure 1 outlines data on patient enrolment, exclusion criteria, and follow-up. The primary endpoint was platelet loss during CRRT. Secondary outcomes were the efficacy of CRRT, measured by steady-state blood urea nitrogen (BUN) during CRRT, the need for platelet substitution (platelet count of less than 20 × 10^9^/L) and major bleeding signs. Major bleeding included any bleeding requiring surgical intervention with a timely connection with CRRT, bleeding documented by computed tomography and/or ultrasound (intracerebral as well as retroperitoneal, abdominal, intestinal, or urogenital) or a decrease in haemoglobin of greater than 5 g/dL within 72 hours with a timely connection with CRRT. Minor bleeding involves a haemoglobin drop of less than or equal to 5 g/dL with or without an identified bleeding site.

CRRT was performed as continuous veno-venous haemodialysis, using a pump system (ADM; Fresenius, Bad Homburg, Germany) and capillary polysulfone haemofilters (Ultraflux^® ^AV 1000S; Fresenius). Blood flow ranged from 100 to 120 mL/hour. Dialysis flow was, on average, 2,000 mL/hour. The ultrafiltration rate was adjusted to patient hydratation and haemodynamic status. Haemofilters and tubing were changed routinely every 24 hours according to the manufacturer's recommendations. Therefore, blood was reinfused to the patient and the entire set of single-use tubes was changed together with the haemofilter. Blood products were administered during a CRRT pause if necessary when haemofilters were changed. The efficacy of CRRT was measured by mean treatment dose and steady-state BUN during CRRT [17]. The study drugs standard unfractioned heparin (UFH) and tirofiban were administered into the extracorporeal circuit as a prefilter infusion. All patients received UFH (Heparin-Natrium-ratiopharm^®^; ratiopharm GmbH, Ulm, Germany) by intravenous bolus application of 80 IU/kg followed by a continuous infusion with 18 IU/kg per hour. For UFH dose titration, plasma activated partial thromboplastin time (aPTT) was measured every hour until a two- to three-fold aPTT was reached. In cases of a steady state, CRRT was started and aPTT was measured twice daily. The short-acting reversible GP IIb/IIIa inhibitor tirofiban (Aggrastat^®^; MSD Sharp & Dohme GmbH, Haar, Germany) has a protein binding of 65% and an elimination half-life of 1.5 to 2 hours predominantly achieved via the renal pathway. According to the manufacturer's recommendations for severe renal insufficiency (creatinine clearance of less than 30 mL/minute), patients of the tirofiban group received, in addition to UFH, tirofiban by intravenous bolus application of 0.2 μg/kg per minute over 30 minutes followed by a continuous infusion with 0.05 μg/kg per minute. According to clinical guidelines, prophylactic platelet transfusions are recommended beyond a platelet count of less than 10 × 10^9^/L [18]. Because of the off-label use of tirofiban, the threshold level for prophylactic platelet transfusion was changed to 20 × 10^9^/L.

### Laboratory tests

To determine changes in haemostasis during the passage of blood through the extracorporeal circuit, blood was sampled in citrate tubes from the efferent line of the extracorporeal circulation (postfilter). All tests were performed in duplicate. Blood samples for analysis of full clinical chemistry, haematology, and platelet-monocyte aggregates were taken before starting CRRT and the following 4 days after starting treatment. The bleeding time was measured by the standardised Ivy method [19]. Other causes of platelet loss were excluded by HIT-II screening tests using the particle gel immunoassay (ID-HPF-4; DiaMed, Cressier, Switzerland) for rapid detection and the enzyme-linked immunosorbent assay for discovering antibodies (IgG, IgA, and IgM) to heparin-platelet factor-4 complexes. Both HIT-II tests were done for all patients. Flow cytometry is a sensitive technique that permits the use of whole blood to assess platelet function in a physiological manner although the interaction of blood with the endothelium is excluded [20]. Staining platelets with antibodies was performed immediately after blood collection, avoiding artificial platelet activation and aggregation. Platelets were identified by monoclonal anti-human antibodies directed against CD41 (clone HIP8, phycoerythrin-conjugated; BD Pharmingen, Heidelberg, Germany), the activated form of GP IIb/IIIa receptors by PAC-1 (clone PAC-1, fluorescein isothiocyanate-conjugated; BD Pharmingen), and monocytes by CD14 (clone RMO52, phycoerythrin-cyanin [PECy5]-conjugated; Beckman Coulter, Krefeld, Germany). Increases in PAC-1 have been shown to be directly correlated with the activation of GP IIb/IIIa binding to fibrinogen and/or monocytes. Measurements were performed by flow cytometer (FACSCalibur; Becton Dickinson, Heidelberg, Germany) and the Cellquest software system (Becton Dickinson, Heidelberg, Germany). Monocytes were selectively gated for analysis by forward scatter, side scatter, and CD14-PECy5. The percentages of PAC-1^+^/CD41a^+^/CD14^+ ^platelet-monocyte aggregates were measured. Nonspecific immunofluorescence was determined using unspecific control monoclonal antibodies.

### Statistical methods

The sample size calculation was performed by the software of the Survey System (Creative Research Systems, Petaluma, CA, USA). The sample size was calculated by the following acceptations: a platelet loss of more than 50% to baseline and a variability of platelet counts of 15%. To detect platelet loss with a power of 95%, a sample size of at least 20 patients in each study group was required. To compare the two treatment regimens, the Mann-Whitney *U *test and analysis of variance were used. Data were given as mean ± standard deviation. Differences were considered significant if the *P *value was less than 0.05. Observed-to-expected (O/E) mortality ratios were reported for each group using the observed-to-SAPS II (Simplified Acute Physiology Score) expected rates per group. Ninety-five percent confidence intervals were calculated.

## Results

Forty patients with cardiogenic shock and acute kidney injuries receiving CRRT were studied for an alternative anticoagulation regimen with the GP IIb/IIIa receptor antagonist tirofiban. Baseline characteristics of the patients are shown in Table 1. All baseline characteristics were well balanced between the treatment groups. Thirty-six of the patients had an acute myocardial infarction, and only four patients had a cardiogenic shock based on acute on chronic heart failure. Clinical procedures are summarised in Table 2. All patients with acute coronary syndromes received a percutaneous coronary intervention, and in 23 cases an IABP was implanted. Most patients received vasoactive therapy at randomisation and during the whole study period of 4 days.

**Table 1:** Demographic and baseline clinical characteristics of patients

	UFH (n = 20)	UFH + tirofiban (n = 20)	*P *value
Demographic data			
Age in years, median (range)	71 (44, 85)	70 (52, 81)	0.932
Female/male, number	8/12	9/11	0.757
Severity of illness scores			
APACHE II score, median (range)	27 (18, 34)	28 (18, 34)	0.523
SAPS II, median (range)	46 (31, 66)	48 (30, 64)	0.768
Cardiogenic shock: reasons and haemodynamics at admission			
Acute coronary syndromes, number	17	19	0.304
Acute decompensation of CHF, number	3	1	0.304
Left ventricular ejection fraction as a percentage, median (range)	31 (20, 57)	30 (18, 54)	0.446
Cardiac index in L/minute per square metre, median (range)	2 (1.4, 2.4)	2 (1.6, 2.4)	0.955
Renal failure: reasons and parameters at admission			
Acute kidney injury, number	16	17	0.688
Acute decompensation of CRI, number	4	3	0.688
Creatinine in mg/dL, mean ± SD	2.9 ± 0.3	2.5 ± 0.2	0.788
Blood urea nitrogen in mg/dL, mean ± SD	72 ± 23.3	70 ± 24.1	0.734
Haematology			
Platelet count, × 10^9^/L, mean ± SD	216 ± 64.3	194 ± 39.5	0.212
Monocyte count, × 10^6^/L, mean ± SD	1,059 ± 85.4	981 ± 103	0.561
Platelet-monocyte aggregates as a percentage, mean ± SD	20.2 ± 5.9	20.8 ± 6.1	0.751

APACHE, Acute Physiology and Chronic Health Evaluation; CHF, chronic heart failure; CRI, chronic renal insufficiency; SAPS, Simplified Acute Physiology Score; SD, standard deviation; UFH, unfractioned heparin.

**Figure 2:**
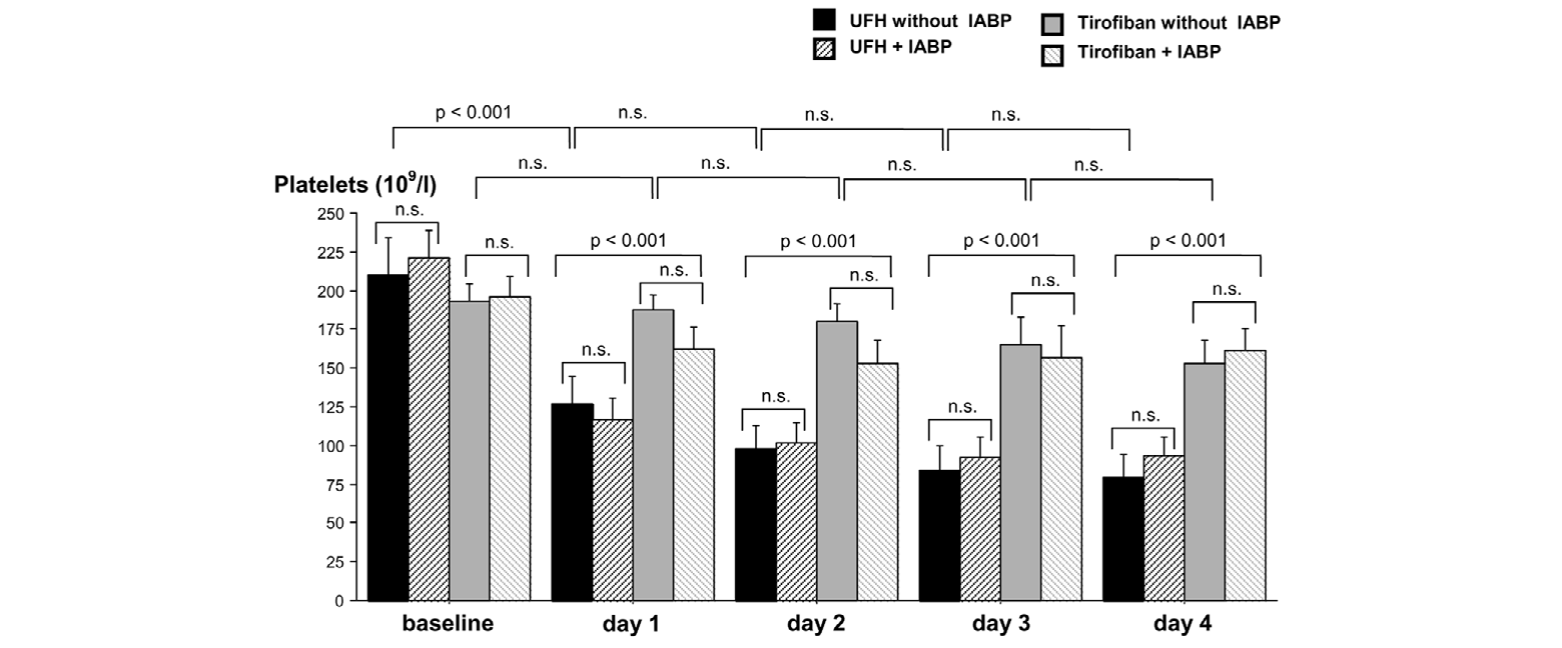
Mean platelet counts during the study period in patients treated with unfractioned heparin (UFH) versus UFH + tirofiban and with or without intra-aortic balloon pump (IABP). Data are shown as mean ± standard deviation. n.s., not significant.

Platelet counts are shown in Figure 2. Baseline platelet counts in the two treatment groups were equivalent (194 ± 39.5 versus 216 ± 64.3 × 10^9^/L, *P *= n.s. [not significant], n = 20). Already after 1 day, patients assigned to tirofiban + UFH had a significant higher platelet count compared with patients assigned to UFH (172 ± 52.9 versus 121 ± 49.2 × 10^9^/L, *P *= 0.003, n = 20). This difference between the two treatment groups continued over the study period up to 4 days (158 ± 45.3 versus 87.3 ± 41.1 × 10^9^/L, *P *< 0.0001, n = 20).

The influence of IABP treatment on platelet count is shown in Figure 2. At day 2, which is the mean IABP duration, there was no significant difference in platelet count between the UFH groups with or without IABP. In the same way, no significant differences could be observed in the tirofiban groups with or without IABP. Similarly, no differences in platelet count in patients with or without IABP were detected on days 3 and 4. After discontinuation of IABP, no significant increase in platelet count was observed for either the UFH or the tirofiban group until the end of the study period.

**Figure 3:**
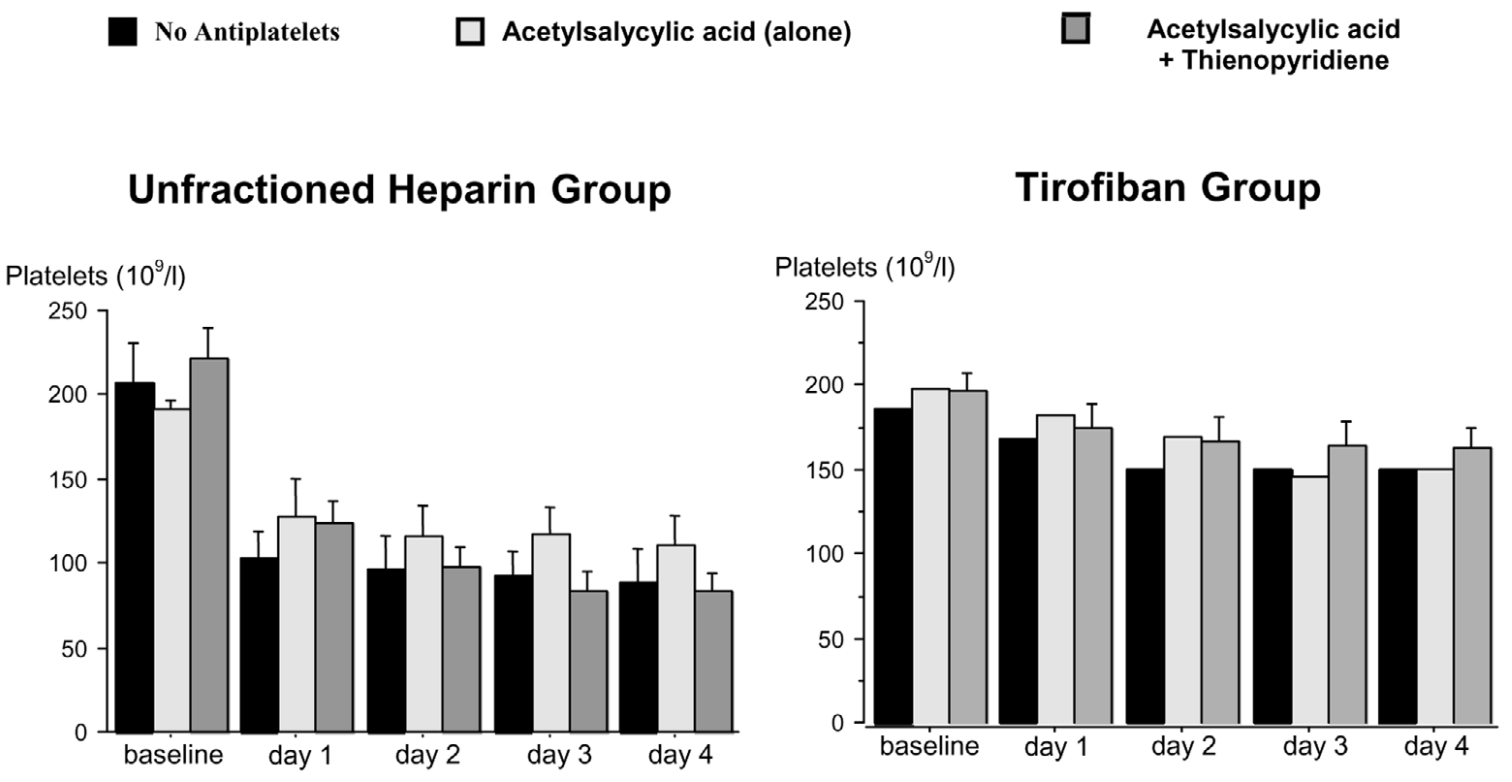
Mean platelet counts during the study period in patients treated with unfractioned heparin (UFH) versus UFH + tirofiban and with different antiplatelet therapy regimens. Data are shown as mean ± standard deviation.

Besides the different anticoagulation with UFH or UFH and tirofiban, the patients were treated with different antiplatelet regimens (no antiplatelets, acetylsalicylic acid alone, or combined antiplatelet therapy with acetylsalicylic acid and thienopyridine) (Table 2). Since the number of patients in the subgroups with no antiplatelet therapy or with acetylsalicylic acid alone was too low, no statistical analysis could be performed. But, as shown in Figure 3, the course of platelet count was comparable in the three antiplatelet subgroups during the whole study period.

**Table 2:** Clinical procedures

	UFH (n = 20)	UFH + tirofiban (n = 20)	*P *value
Cardiac procedures			
Coronary angiography, number	18	19	0.560
Percutaneous coronary intervention, number	15	18	0.560
Intra-aortic balloon pump, number	11	12	0.876
Intra-aortic balloon pump duration in hours, mean ± SD	48 ± 14.4	50 ± 12.5	0.757
Haemodialysis characteristics			
Treatment dose in mL/kg per hour, mean ± SD	28 ± 2.5	28 ± 2.9	0.381
Blood urea nitrogen (BUN)			
Pretreatment BUN in mg/dL, mean ± SD	72 ± 23.3	70 ± 24.1	0.734
Steady-state BUN during CRRT in mg/dL, mean ± SD	32 ± 18.1	31 ± 22.1	0.734
Antiplatelet therapy and anticoagulation			
No antiplatelets, number	3	1	-
Acetylsalicylic acid alone, number	2	1	-
Acetylsalicylic acid and thienopyridine, number	15	18	-
UFH, number (dose in IU/kg per hour, mean ± SD)	20 (18.4 ± 0.6)	20 (18.2 ± 0.8)	0.872
Activated partial thromboplastin time in seconds, mean ± SD	64 ± 13.2	62 ± 11.8	0.621
Ivy bleeding time in seconds, mean ± SD	422 ± 58.1	599 ± 118.1	0.003
Further concomitant therapy			
Dobutamine, number (dose in μg/kg per minute, mean ± SD)	18 (6 ± 2.8)	19 (6 ± 3.2)	0.560
Norepinephrine, number (dose in μg/kg per minute, mean ± SD)	14 (0.2 ± 0.1)	13 (0.2 ± 0.15)	0.744
Opioids and benzodiazepins, number	12	14	0.519
Mechanical ventilation, number	12	14	0.519

CRRT, continuous renal replacement therapy; SD, standard deviation; UFH, unfractioned heparin.

The efficacy of CRRT was estimated by mean treatment dose, steady-state BUN during CRRT, and haemofilter life span. The calculated mean treatment dose was 25 to 30 mL/kg per hour in both anticoagulation regimens and confirmed by an acceptable steady-state BUN during CRRT (Table 2).

The platelet transfusion threshold was defined as a platelet count of less than 20 × 10^9^/L. During the study period, in three patients of the UFH group, a platelet loss of less than 20 × 10^9^/L was registered without any bleeding signs. Two patients received one platelet unit and one patient needed two units for platelet increase. The number of platelet units related to the cumulative days of CRRT was calculated as 0.05 ± 0.02. In the tirofiban group, no platelet transfusion was necessary. Thus, the difference of platelet transfusions between the two groups was significant (*P *= 0.016, n = 20) (Table 3).

**Table 3:** Primary and secondary endpoints

	UFH (n = 20)	UFH + tirofiban (n = 20)	*P *value
Platelet/Monocyte counts at the end of CRRT			
Platelet count, × 10^9^/L, mean ± SD	87 ± 41.1	158 ± 45.3	0.001
Monocyte count, × 10^6^/L, mean ± SD	945 ± 77.3	1,394 ± 151	0.012
Platelet-monocyte aggregates as a percentage, mean ± SD	27.5 ± 9.3	3.9 ± 2.1	0.001
Bleeding events during CRRT			
Minor bleeding, number	2	1	0.560
Major bleeding, number	0	0	1
Platelet transfusions during CRRT			
Platelet units per patient per day, mean ± SD	0.05 ± 0.02	0	0.016
Outcome			
Intensive care unit mortality rate, number (percentage)	8 (40)	7 (35)	0.752
Hospital mortality rate, number (percentage)	8 (40)	7 (35)	0.752
SAPS II predicted mortality rate as a percentage	36.9	41.4	-
Observed-to-expected mortality ratio	1.08	0.85	-
95% confidence interval for the observed-to-expected mortality ratio	0.46, 1.97	0.34, 1.59	-

Values are presented as number of patients or mean ± standard deviation (SD). CRRT, continuous renal replacement therapy; SAPS, Simplified Acute Physiology Score; UFH, unfractioned heparin.

The study was not powered for mortality. The in-hospital mortality rates were 35% in the UFH + tirofiban group and 40% in the UFH group. ICU mortality, hospital mortality, O/E mortality ratios, and 95% confidence intervals were calculated (Table 3).

**Figure 4:**
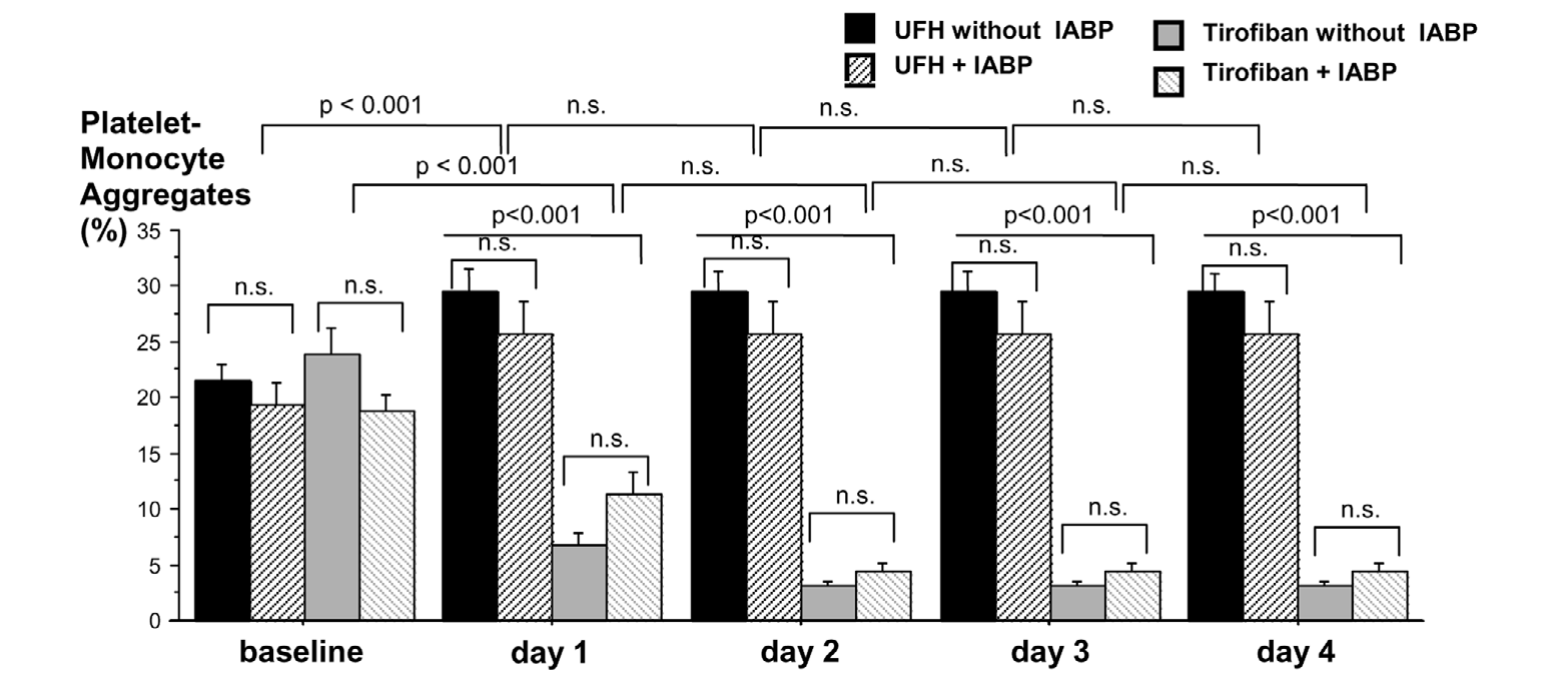
Mean platelet-monocyte aggregates during the study period in patients treated with unfractioned heparin (UFH) versus UFH + tirofiban and with or without intra-aortic balloon pump (IABP). Data are shown as mean ± standard deviation. n.s., not significant.

In all patients, the baseline levels of monocytes were not different between the two anticoagulation regimens (UFH + tirofiban versus UFH: 981 ± 103.6 versus 1,059 ± 85.4 × 10^6^/L, n = 20, *P *= n.s.). During CRRT with UFH + tirofiban, monocyte counts increased significantly (1,394 ± 151 versus 945 ± 77.3 × 10^6^/L, n = 20, *P *= 0.012). The percentage of PAC-1/CD41a-positive monocytes before starting CRRT was equivalent between the two anticoagulation regimens (20.8% ± 6.1% versus 20.2% ± 5.9%, n = 20, *P *= n.s.). Within 24 hours, the combined UFH + tirofiban anticoagulation resulted in a decrease of PAC-1/CD41a-positive platelet-monocyte coaggregates whereas with UFH alone these coaggregates increased (9.5% ± 5.8% versus 27.5% ± 9.3%, n = 20, *P *< 0.001). The follow-up of 4 days presented a further decrease of platelet-monocyte coaggregates in the tirofiban group; within the UFH group, the coaggregates remained stable but elevated to baseline (27.5% ± 9.3% versus 20.2% ± 5.9%, n = 20, *P *< 0.001). This difference between the two anticoagulation regimens was already present after 24 hours of treatment (Figure 4).

## Discussion

In a pilot study, we investigated the possible use and effectiveness of the reversible platelet GP IIb/IIIa receptor inhibitor tirofiban to preserve platelet number and function during CRRT in patients with cardiogenic shock. Tirofiban additional to UFH for anticoagulation apparently prevented platelet loss over a period of 96 hours of CRRT. Furthermore, the inhibition of the activated platelet fibrinogen receptor GP IIb/IIIa (PAC-1) by tirofiban results in an inhibition of platelet-leukocyte interaction and aggregation [7,8,21]. We examined changes in platelet loss and platelet-monocyte coaggregates by analysing the platelet-specific CD41a and PAC-1 antigen on monocytes using three-color flow cytometry as whole-blood technique. The percentage of platelet-monocyte coaggregates showed a highly significant decrease by combined anticoagulation with UFH and tirofiban. Platelet-monocyte aggregates were shown to promote monocyte adhesion to endothelium and to induce proinflammation [22-25]. Our findings suggest that the combined anticoagulation with UFH and tirofiban during CRRT inhibits platelet activation and platelet-monocyte interactions with consequences for platelet protection and antithrombotic and anti-inflammatory effects. In contrast, the treatment with UFH alone increased platelet-monocyte binding.

Platelet loss under CRRT in the UFH group was marked. This may be related to the critically ill patients with cardiogenic shock combined with acute kidney injury. Our results are comparable to other examinations of critically ill patients with multiple organ dysfunction syndrome and acute kidney injuries [26]. Neither the concomitant treatment with intra-aortic counterpulsation nor the antiplatelet therapy with acetylsalicylic acid and thienopyridine had an effect on the platelet loss between these subgroups.

The efficacy of CRRT assessed by mean treatment doses and steady-state BUN during CRRT was comparable in the two treatment groups. Despite the different anticoagulation regimens and the higher potency of anticoagulation within the tirofiban + UFH group, this therapy was not associated with an increased number of bleeding events. To minimise the risk of bleeding, tirofiban and UFH were administered into the extracorporeal circuit as a prefilter infusion. The study was not focused on bleeding events and therefore an analysis regarding bleeding events would be totally underpowered. But as a result, no clinically important bleedings were detected and no transfusions of red blood cells or platelet units were necessary in patients treated with the combined tirofiban + UFH anticoagulation. Nevertheless, further studies are warranted to ascertain the safety of an anticoagulation regimen with tirofiban + UFH during long-term CRRT.

One might argue that the study could be limited by (a) the open-label character of its design, (b) the small number of patients, (c) the lack of a specific antidote for tirofiban, and (d) missing data on long-term efficacy and bleeding events of tirofiban during CRRT. Because of the pilot-study character and the off-label use of tirofiban during CRRT, the physicians were not blinded. However, clinical evaluation and determination of primary endpoints were done separately by clinical and experimental investigators, the latter of which were blinded to the clinical data of the patients. As there is no specific antidote for tirofiban in cases of bleeding events, donor platelets should be transfused and haemofiltration is suggested for extracorporeal elimination of tirofiban [27]. A recent development of a rapid whole-blood point-of-care platelet function assay, the rapid platelet function assay, now allows for the bedside monitoring of platelet inhibition by GP IIb/IIIa receptor antagonists [28]. Further investigations with larger numbers of patients are necessary for the determination of haemofilter run times, long-term efficacy, and bleeding events of tirofiban during CRRT.

## Conclusion

The GP IIb/IIIa receptor antagonist tirofiban inhibits platelet activation and platelet-monocyte interaction. Its use in addition to UFH during CRRT prevents platelet loss and preserves platelet function.

## Key messages

• The glycoprotein IIb/IIIa receptor antagonist tirofiban inhibits platelet activation and platelet-monocyte interaction.

• The use of tirofiban during continuous renal replacement therapy prevents platelet loss and preserves platelet function.

## Abbreviations

aPTT: activated partial thromboplastin time; BUN: blood urea nitrogen; CRRT: continuous renal replacement therapy; GP: glycoprotein; HIT-II: heparin-induced thrombocytopenia type II; IABP: intra-aortic balloon pump; ICU: intensive care unit; n.s.: not significant; O/E: observed-to-expected; PAC-1: activated platelet fibrinogen receptor glycoprotein IIb/IIIa; PECy5: phycoerythrin-cyanin; UFH: unfractioned heparin.

## Competing interests

The authors declare that they have no competing interests. This study, which originally included 20 patients, was initiated with financial support from MSD Sharp & Dohme GmbH. Investigations of an additional 20 patients were financed by the authors.

## Authors' contributions

AL helped to initiate the study, participated in the statistical analysis of the data and in interpreting the data, and drafted the manuscript. MG led CRRT and participated in the statistical analysis of the data and in interpreting the data. SS and RR participated in experimental investigations. MB helped to initiate the study and participated in the statistical analysis of the data and in interpreting the data. All authors read and approved the final manuscript.
